# Enhancing Dose Efficiency of Optimum Bright‐Field Scanning Transmission Electron Microscopy Using a Phase‐Shifted Electron Probe

**DOI:** 10.1002/smtd.202501661

**Published:** 2026-05-17

**Authors:** Mitsuru Nogami, Takehito Seki, Kousuke Ooe, Naoya Shibata

**Affiliations:** ^1^ Institute of Engineering Innovation School of Engineering The University of Tokyo Bunkyo‐ku Tokyo Japan; ^2^ PRESTO Japan Science and Technology Agency Kawaguchi Japan; ^3^ School of Physics and Astronomy Monash University Clayton Victoria Australia; ^4^ Nanostructures Research Laboratory Japan Fine Ceramics Center Nagoya Aichi Japan

**Keywords:** optimum bright field, ptychography, scanning transmission electron microscopy

## Abstract

Observing beam‐sensitive materials using scanning transmission electron microscopy (STEM) at low electron dose conditions is challenging due to the low signal‐to‐noise ratio (SNR) of images. In this study, we aimed to improve the SNR of optimum bright‐field (OBF) STEM by introducing phase shifts to the incident electron probe using lens aberrations. Theoretical analysis and multislice simulations revealed that spherical aberration enhances low‐frequency contrast without introducing image artifacts, unlike simple defocus, which causes oscillations in the contrast transfer function. This approach improves the visibility of structural features in low‐dose conditions and may enable more efficient imaging of beam‐sensitive materials such as zeolites.

## Graphical abstract

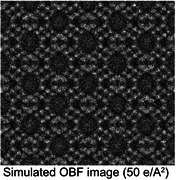

## 1 Introduction

Scanning transmission electron microscopy (STEM) is a powerful imaging technique that enables atomic resolution observation of local structures of materials. STEM can be applied to various kinds of observation targets such as grain boundaries, surfaces, and point defects [1]. On the other hand, it has been difficult to observe some kinds of materials using STEM. Beam‐sensitive materials are one of the representatives of such materials. During the STEM observation, the specimen is illuminated by a high‐energy electron beam. Depending on the material, this beam can cause damage either by knock‐on displacement or by inelastic processes such as radiolysis [2]. To avoid the specimen structure from being destroyed, the electron dose has to be limited. However, limited electron dose results in low signal‐to‐noise ratio (SNR) of the image, where it is difficult to analyze specimen structure in detail. Beam‐sensitive materials include industrially important materials such as battery materials, zeolites, and metal‐organic frameworks. Therefore, a STEM imaging technique that is capable of visualizing beam‐sensitive material at low‐dose is demanding.

Recently, new types of STEM detector, such as pixelated detector [3, 4] and segmented detector [5], were developed. Accordingly, new types of imaging techniques are developed using these detectors and are expected to be effective for observing beam‐sensitive materials at low‐dose conditions.

One of the major applications of pixelated detector is electron ptychography [6, 7, 8]. Electron ptychography reconstructs the complex transmission function from 4D dataset [9] recorded by pixelated detector. Because of the redundancy of 4D dataset, electron ptychography is a dose‐efficient imaging technique [10] and can be applied to the observation of beam‐sensitive materials at low‐dose conditions [11, 12, 13].

Another dose‐efficient STEM imaging technique is optimum bright‐field (OBF) STEM [14, 15]. OBF is a non‐iterative method to calculate image from dataset obtained by segmented/pixelated detectors. The calculation method is designed to maximize the SNR of the resulting image under the weak phase object approximation (WPOA). By using thick weak phase object approximation (tWPOA) [16, 17], OBF can be applied to specimens with nonnegligible thicknesses. OBF is a highly sensitive imaging technique and is demonstrated to be effective for visualizing beam‐sensitive materials at low‐dose conditions [18].

OBF is the optimum reconstruction process of images under the tWPOA. However, preferred conditions at the time of image acquisition have not been investigated. It has been reported that modulating the phase of the incident electron by a phase plate or defocus can enhance the image signal using electron ptychography [19]. Therefore, it can be considered that the SNR of OBF images may also be enhanced by modulating the phase of incident electron at the time of dataset acquisition.

In this study, the relationship between SNR of OBF images and phase shift of incident electron was theoretically investigated. As a result of noise normalized PCTF [20] calculation and multislice image simulation, SNR of low spatial frequency component of OBF image for relatively thin specimen can be enhanced by lens aberrations. Especially, spherical aberration can enhance PCTF while keeping interpretable contrast‐transfer characteristics.

## 2 Theory

This section describes how lens aberrations enhance the signal to be recorded by the STEM pixelated detector. In the first part, we discuss under the weak‐phase object approximation (WPOA). In the latter part, discussion is extended to the condition where specimen thickness cannot be ignored.

### 2.1 Weak Phase Object Approximation

Under the WPOA, it has been reported that the contrast of STEM image recorded by pixelated detectors can be enhanced by modulating the phase of the incident electron [19]. In STEM, intensity recorded by a pixelated detector can be expressed as *I*(***k***, *** R***_***p***_), where ***R***_***p***_ and ***k*** represent probe position and the coordinate on the detector plane, respectively. Under the weak phase object approximation, Fourier transform of *I*(***k***, *** R***_***p***_) with respect to probe position ***R***_***p***_ can be expressed as follows [7]:

(1) $$\begin{array}{ccc} G\left(k,\;Q_{p}\right) & = & {\left|T\left(k\right)\right|}^{2}\delta \left(Q_{p}\right)+i\left[T^{*}\left(k\right)T\left(k-Q_{p}\right)\right.\\ & & -\left.T\left(k\right)T^{*}\left(k+Q_{p}\right)\right]\sigma V\left(Q_{p}\right), \end{array}$$

where *T*(***k***) = *A*(***k***)exp (− iχ(***k***)) is a lens transfer function. *A*(***k***) is the aperture function. The aperture function has a constant value *A*_0_ inside the aperture (| ***k*** | < *k*_0_, *k*_0_ denoting the radius of the aperture) and 0 outside. *A*_0_ is a constant such that $\int {\left|A(k)\right|}^{2}dk=1$. $\chi \left(k\right)=\frac{2\pi }{\lambda }(\frac{1}{4}C_{s}\lambda^{4}|k{|}^{4}-\frac{1}{2}\Delta f\lambda^{2}\left|k{|}^{2}\right)+\chi^{\prime }\left(k\right)$ is the aberration function where λ is the electron wavelength, *C_s_* is spherical aberration coefficient, Δ*f* is defocus value, and χ′(***k***) is the aberration function other than the spherical aberration and defocus. σ is the interaction parameter. *V*(***Q***_***p***_) is the Fourier transform of the projected potential of the specimen. Information on specimen structure *V*(***Q***_***p***_) is transferred to the second term of Equation (1).

**FIGURE 1:**
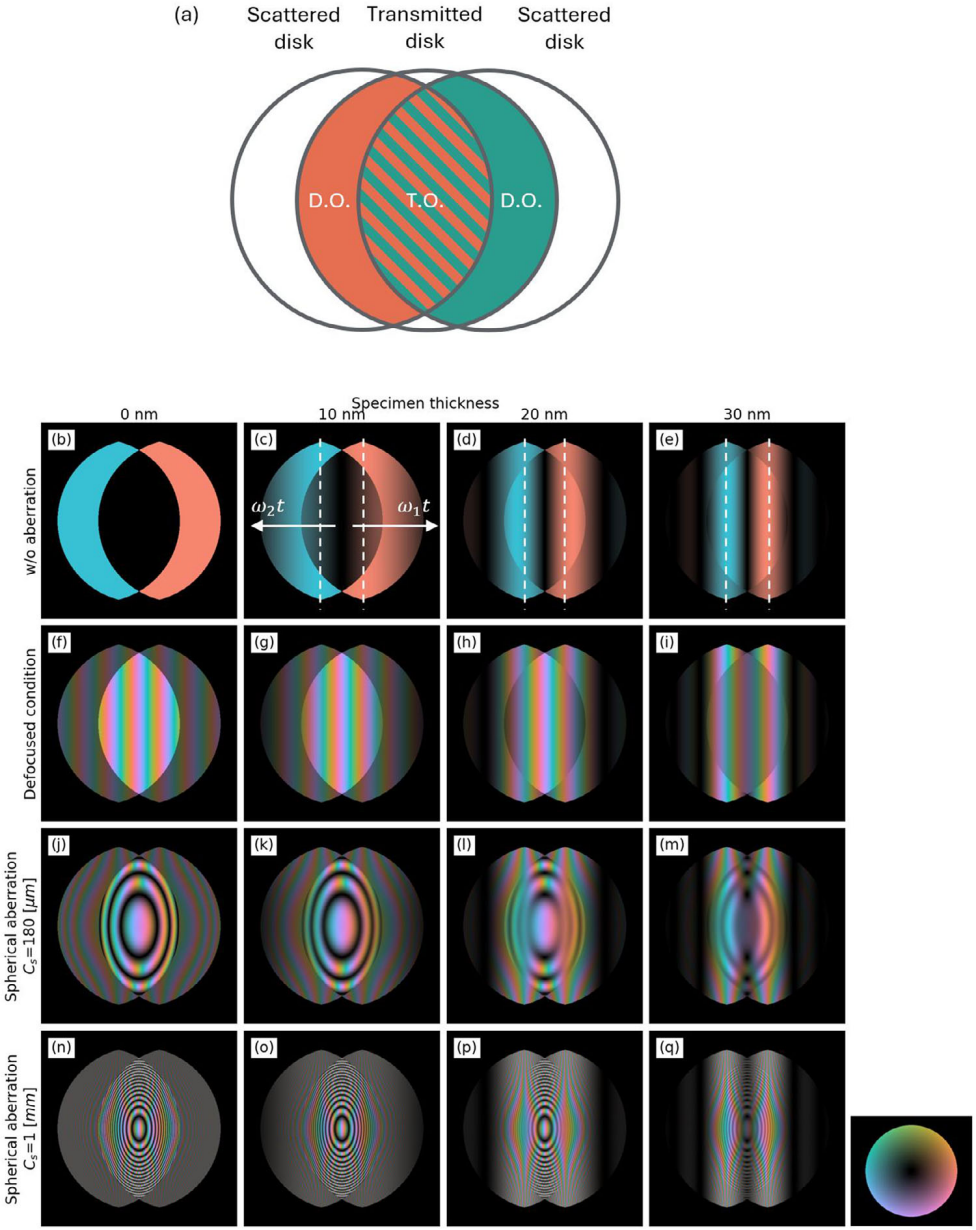
Analysis of disk overlap patterns. (a) Schematic diagram of the overlap between the transmitting and scattering disks. Disk overlap patterns calculated based on either Equations (1) or (2), for the thicknesses of 0, 10, 20 and 30 nm: (b–e) without aberrations, (f–i) with defocus Δf = 30 nm, (j–m) with spherical aberration Cs = 180 µm, and (n–q) with spherical aberration Cs = 1000 µm. Complex values are visualized using the phase–amplitude perceptually uniform colormap (PAPUC) [21].

For a specific spatial frequency $Q_{p}\;\left(Q_{p}\neq 0\right)$, Equation (1) can be considered as overlap of three disks: transmitted disk centered at ***k*** = 0 and two disks deflected to ***k*** = ± ***Q***_***p***_ **. $\Gamma_{+Q_{p}}=T^{*}\left(k\right)T\left(k-Q_{p}\right)$ term expresses interference between the transmitted disk and the disk deflected to + ***Q***_***p***_. We denote this term as $\Gamma_{+Q_{p}}$. This term has non‐zero value only at the region where these two disks overlap. Likewise, $\Gamma_{-Q_{p}}=-T\left(k\right)T^{*}\left(k+Q_{p}\right)$ expresses the interference between the transmitted disk and the disk deflected to − ***Q***_***p***_ and has non‐zero value only at the region where two disks overlap. For small ***Q***_***p***_ (| ***Q***_***p***_| < *k*_0_), overlap of disks can be classified into two regions, double overlap (D.O.) and triple overlap (T.O.) regions, as shown in Figure 1a. In the D.O. region, only one of $\Gamma_{+Q_{p}}$ and $\Gamma_{-Q_{p}}$ has non‐zero value. In the T.O. region, both $\Gamma_{+Q_{p}}$ and $\Gamma_{-Q_{p}}$ have non‐zero value. If incident electron has flat phase, i.e., no aberration, $\Gamma_{+Q_{p}}$ and $\Gamma_{-Q_{p}}$ has a constant complex value with same amplitude and opposite phase, resulting in *G*(***k***, *** Q***_***p***_) = 0 in the T.O. region [10] as shown in Figure 1b. Therefore, information of the specimen potential *V*(***Q***_***p***_) is not transferred to the T.O. region. This is the reason why the contrast transfer is poor for the low‐spatial frequency region when using a focused probe. On the other hand, when the phase of incident electron is not flat due to the presence of lens aberrations, generally $G(k,\;Q_{p})\neq 0$. Figure 1f,j,n show the *G*(***k***, *** Q***_***p***_) in the presence of aberration. Disk overlap pattern has non‐zero value in the T.O. region. Thus, information of the specimen structure *V*(***Q***_***p***_) is transferred to broader region. Therefore, by modulating the phase of incident electron, information about specimen may be more effectively retrieved from the *G*(***k***, *** Q***_***p***_).

### 2.2 Thick Weak‐Phase Object Approximation

Weak phase object approximation ignores the specimen thickness and does not consider the propagation of incident electron inside the specimen. This assumption is valid if the specimen thickness is small enough compared to the depth of field of electron probe. However, under typical condition for atomic resolution observation, specimen thickness may be thicker than the depth of field of the electron probe. Here, the previous discussion is extended to the condition where the specimen thickness is not negligible. To consider finite specimen thickness effect, thick weak‐phase object approximation (tWPOA) was adopted in this paper.

Under the tWPOA, specimen with finite thickness is sliced into thin slices along the beam direction. Each slice is assumed to be a weak phase object. In addition, multiple scattering is ignored. Under these assumptions, the total signal to be recorded by a pixelated detector can be considered as sum of signals independently scattered by each slice. For $Q_{p}\neq 0$, Fourier transform of signal to be detected with respect to the probe position can be expressed as follows:

(2) $$\begin{array}{ccc} G\left(k,\;Q_{p}\right) & = & i\sum_{j}\left[T^{*}\left(k,\;z_{j}\right)T\left(k-Q_{p},\;\;z_{j}\right)\right.\\ & & -\left.T\left(k,\;z_{j}\right)T^{*}\left(k+Q_{p},\;z_{j}\right)\right]\sigma V_{j}\left(Q_{p}\right), \end{array}$$

*z_j_* is the position of *j*‐th slice along beam direction. The mid‐plane of the specimen is defined as *z_j_* = 0 and the defocus value, Δf, is defined with reference to this mid‐plane position. *T*(***k***,  *z_j_*) = *T*_0_(***k***)exp (− iπλ| ***k*** |^2^ *z_j_*) is the modified lens transfer function with the defocus value at the *j*‐th slice position (*T*_0_(***k***) denoting the lens transfer function at *z* = 0). *V_j_* (***Q***_***p***_) is the Fourier transform of projected potential of the *j*‐th slice. When specimen has periodic structure along *z* direction, specimen can be sliced in the way such that each slice has same projected potential *V_j_* (***Q***_***p***_) = *V*(***Q***_***p***_)/*n* where *n* is the number of slices. Additionally, if the slice is thin enough, sum in Equation (2) can be considered as integral as follows:

(3) $$\begin{array}{ccc} G\left(k,\;Q_{p}\right) & = & \frac{i}{t}\underset{-\frac{t}{2}}{\int^{\frac{t}{2}}}\left[T^{*}\left(k,\;z\right)T\left(k-Q_{p},\;z\right)\right.\\ & & -\left.T\left(k,\;z\right)T^{*}\left(k+Q_{p},\;z\right)\right]\sigma V\left(Q_{p}\right)dz, \end{array}$$

where *t* is the specimen thickness. The integration interval is from $-\frac{t}{2}$ to $\frac{t}{2}$ because the mid‐plane of the specimen is defined to be *z* = 0. Equation (3) can be calculated as follows:

(4) $$G\left(k,\;Q_{p}\right)=i\left[\mathrm{sinc}\left(\omega_{1}t\right)\cdot \Gamma_{+Q_{p}}+\mathrm{sinc}\left(\omega_{2}t\right)\cdot \Gamma_{-Q_{p}}\right]\sigma V\left(Q_{p}\right),$$

where sinc(*x*) = sin (*x*)/*x* when x≠0(sinc(0)=1), $\omega_{1}=\pi \lambda \left(k\cdot Q_{p}-\frac{|Q_{p}{|}^{2}}{2}\right)$, $\omega_{2}=-\pi \lambda \left(k\cdot Q_{p}+\frac{|Q_{p}{|}^{2}}{2}\right)$. Equation (4) is a form similar to Equation (1), but with $\Gamma_{\pm Q_{p}}$ terms multiplied by $\mathrm{sinc}\left(\omega_{i}t\right)\;\left(i\in \{1,\;2\}\right)$. This difference is described in Figure 1b–e with thicknesses of 0, 10, 20, and 30 nm, respectively, in the absence of aberration. In D.O. regions, sinc(ω_*i*_ *t*) represents amplitude oscillation along ***Q***_***p***_ direction on the ***k*** plane, since ω_*i*_ exhibits a dependence on ***k*** · ***Q***_***p***_ as in the definition. ω_*i*_ takes 0 on the perpendicular bisector of the origin (***k*** = 0) and the center of scattered disk (***k*** = ± ***Q***_***p***_) as indicated by the white dashed line in Figure 1c–e, where sinc(ω_*i*_ *t*) has the maximum value. The frequency of sinc(ω_*j*_ *t*) is proportional to the specimen thickness t. In the T.O. regions shown in Figure 1b,c, disk overlap pattern has weak signal. This is because $\mathrm{sinc}\left(\omega_{1}t\right)\cdot \Gamma_{+Q_{p}}$ and $\mathrm{sinc}\left(\omega_{2}t\right)\cdot \Gamma_{-Q_{p}}$ with small *t* have opposite phase and close amplitude. Thus, the low‐frequency components of specimen structure cannot be retrieve from the T.O. region effectively in the absence of lens aberrations. Figure 1f,g,j,k,n,o shows disk overlap pattern for thicknesses of 0 and 10 nm, respectively, in the presence of aberration. By comparing those without aberration, signal in the T.O. regions is enhanced by aberration. Therefore, low‐frequency specimen structure components may be retrieved more efficiently by introducing lens aberrations when specimen thickness is relatively small.

Figure 1d,e shows the disk overlap pattern in the absence of aberration for thicknesses of 20 and 30 nm, respectively. Even without aberration, the T.O. region has strong signal. This is because when the specimen thickness *t* is large, $\mathrm{sinc}\left(\omega_{1}t\right)\cdot \Gamma_{+Q_{p}}$ and $\mathrm{sinc}\left(\omega_{2}t\right)\cdot \Gamma_{-Q_{p}}$ don't have close amplitude or opposite phase. The signal in the T.O. region with the presence of aberration (Figure 1h,i,l,m,p,q) is strong, but the enhancement is less apparent than those for thin specimen case. Therefore, lens aberrations would be less effective to enhance low‐frequency information when the specimen thickness is large.

## 3 Results

### 3.1 SNR Transfer Calculation

To compare the dose efficiency of OBF STEM with and without lens aberrations, phase contrast transfer functions (PCTFs) were calculated under noise‐normalization condition [19], which is referred as to SNR transfer function in the present paper. In the calculation, accelerating voltage was set to 300 kV, convergence semi‐angle was set to 20 mrad, and the specimen thicknesses were set to 0, 10, and 30 nm. The following aberration conditions were compared: aberration free condition, defocus of 30 nm, spherical aberration of 20, 40, 180 and 1000 µm. For the aberration‐free condition, electron probe is assumed to be focused on the mid‐plane of the specimen. As for the defocused condition, electron probe was assumed to be focused on the plane 30 nm away from the mid‐plane of the specimen. For the STEM detector, pixelated detector is assumed.

**FIGURE 2:**
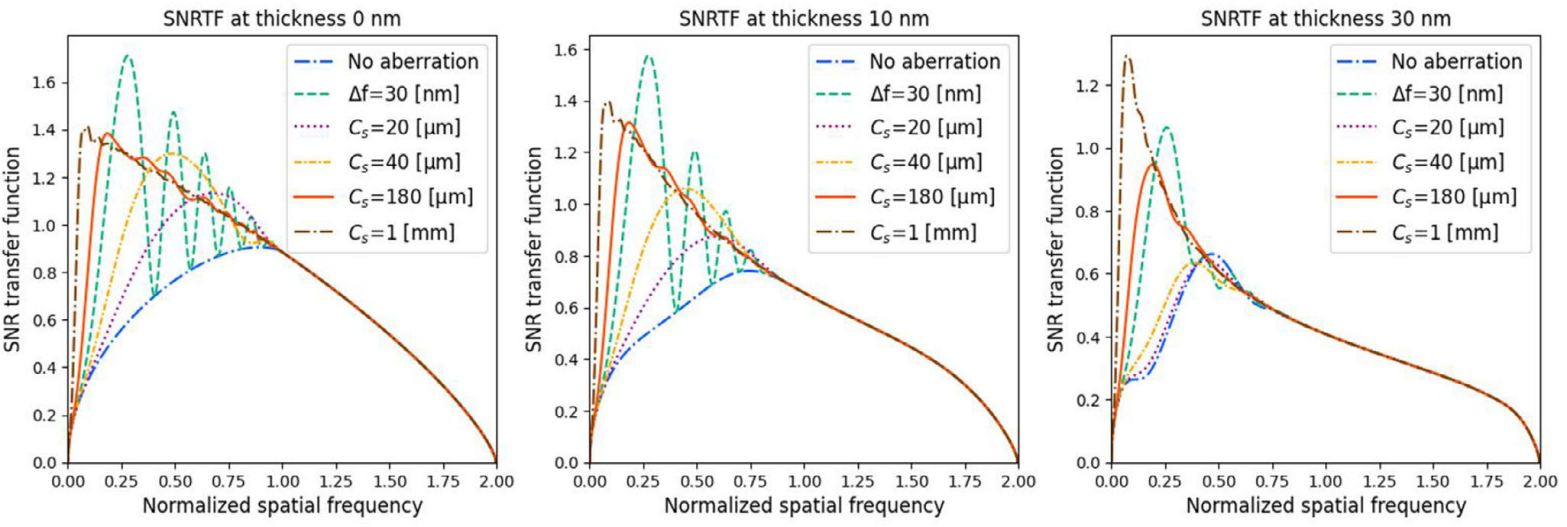
SNR transfer functions for thicknesses of 0, 10, and 30 nm under various aberration conditions.

Figure 2 shows the calculated SNR transfer functions. Under the aberration‐free condition, SNR transfer is lowest in the low spatial frequency region. Defocus and spherical aberration both enhanced SNR transfer in the low spatial region, especially for thin specimens. With defocus, PCTF oscillates in the low spatial frequency region and was only enhanced at specific spatial frequencies. This oscillation may make image interpretation difficult. On the other hand, spherical aberrations enhanced PCTFs while keeping interpretability of images. In principle, increasing Cs enhances the SNR transfer. Nevertheless, the phase fringes in the disk overlap patterns become increasingly finer (Figure 1), complicating practical experimentation. In this paper, we have selected Cs = 180 µm as a practical value. In the high‐frequency region, the SNR transfer functions are identical over all aberration conditions, since there is no T.O. region. In general, introducing aberration in conventional STEM imaging broadens the probe and degrades the resolution. In our OBF scheme, however, the maximum transferred spatial frequency (i.e., the nominal resolution) is essentially unchanged. We further investigated the conditions with a combination of defocus and spherical aberration, and determined that this combination is not particularly effective. Details are provided in the Supporting Information.

### 3.2 Multislice Image Simulation

OBF images were calculated using multislice image simulation under three aberration conditions: aberration‐free condition, defocus of 30 nm, and spherical aberration of 180 µm. The thickness was considered to be either 10 or 30 nm. Faujasite zeolite (FAU) [110] zone‐axis condition was considered as a model sample here. The multislice simulation package *abTEM* [22] was used to simulate signals to be measured by pixelated detector. The scan step was set to 0.25 Å. To consider the effective source size effect, Gaussian blur was applied along the probe position of the simulated dataset. The full width at half maximum of the Gaussian is 0.6 Å. Noise normalization [23] was applied to the OBF image. The other parameters required in the simulation were set the same as in the calculation of SNR transfer function. To perform low‐dose image simulations, we first calculated the expected number of electrons detected on each detector pixel at each probe position from the infinite‐dose 4D datasets. We then generated noisy 4D datasets by sampling from Poisson distributions whose expectation values were obtained in the previous step. Finally, low‐dose simulation images were reconstructed from these noisy 4D datasets.

**FIGURE 3:**
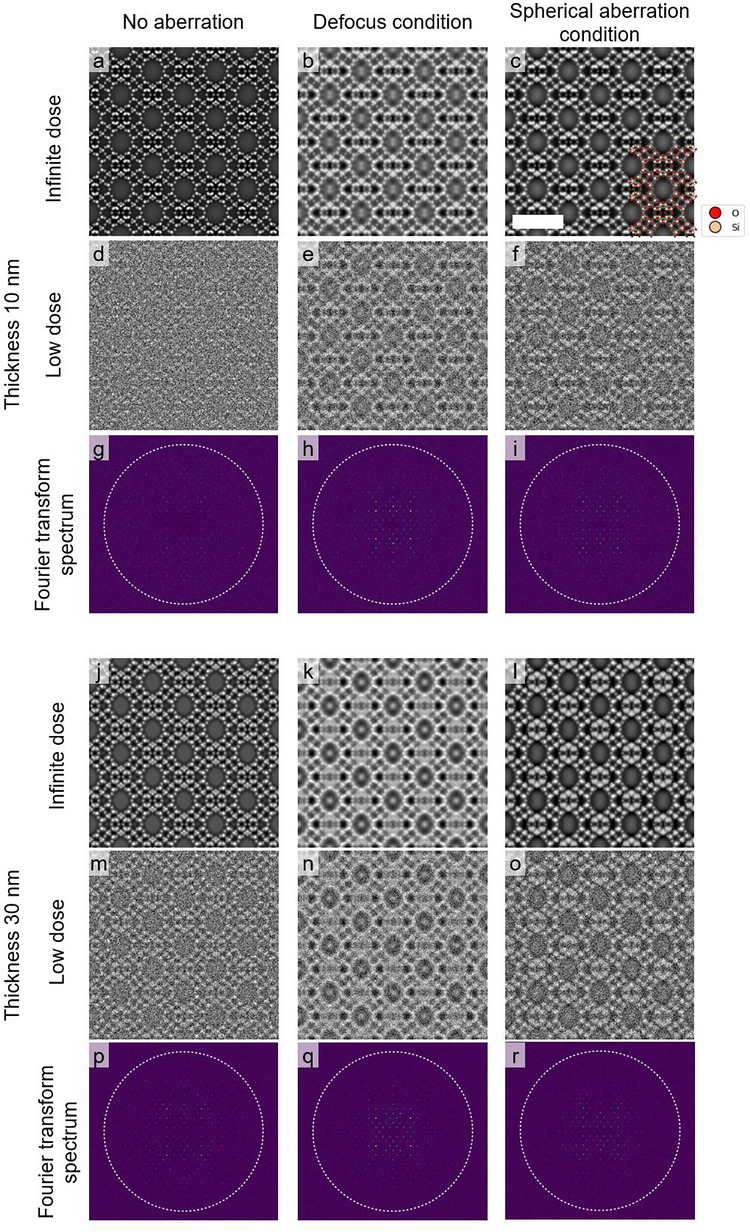
Simulated OBF STEM images of FAU zeolite viewed along [110] zone axis. (a–c) Infinite‐dose images: (a) aberration‐free, (b) defocus of 30 nm, and (c) spherical aberration of 180 µm. (d–f) Corresponding finite‐dose images with 50 e/Å^2^ (equivalent to 3 e/pixel). (g–i) Power spectra of images (d), (e, f), respectively. (j–r) Same as (a–i), but for a 30 nm‐thick specimen: (j–l) infinite‐dose, (m–o) finite‐dose at 50 e/Å^2^, and (p–r) power spectra of (m–o). The scale bar denotes 2 nm. The inset in (c) shows the atomic model (Si and O). The dashed circles in the power spectra indicate the information limit.

To investigate the contrast transfer characteristics, OBF images were simulated with infinite electron dose (Figure 3a–c,j–l). As expected by the SNR transfer functions, OBF image with spherical aberration properly reflects the atomic structure of FAU zeolite without any loss in resolution, maintaining a quality comparable to that observed under aberration‐free conditions. On the other hand, in the OBF image under defocused conditions, contrast that does not reflect true structure, such as showing additional contrasts inside the pores (see Supporting Information for further analysis). These are artifacts due to the oscillation of the SNR transfer function of the defocused condition. Therefore, spherical aberration is considered to be better than defocus in terms of interpretability of OBF image.

To confirm the SNR enhancement effect by introducing aberrations, OBF images were simulated under low‐dose conditions of 50 e^−^/Å^2^ (Figure 3d–f,m–o). Under the aberration‐free condition for the thickness of 10 nm, it is difficult to observe the atomic structure or even the framework of FAU zeolite (Figure 3d). On the other hand, the framework of the specimen is recognizable under the presence of aberrations: defocus and spherical aberration (Figure 3e,f). However, in the defocused condition, the artifact is still visible. Thus, the spherical aberration condition is favorable to enhance SNR of OBF image. Figure 3g–i shows the power spectra of OBF images at the low‐dose condition with the noise level normalized to be identical. These results also show better SNR transfer in the presence of aberrations. For the 30‐nm‐thick case shown in Figure 3j–r, the results are similar to those at 10 nm, except that the SNR‐transfer enhancement is smaller. We compared the disk‐overlap patterns obtained from multislice simulations with those calculated within the tWPOA (Figure S5). The close agreement indicates the tWPOA remains a reasonably good approximation for forming OBF images even for a 30 nm‐thick specimen, despite the dynamical scattering effect. From these results, it can be considered that spherical aberration is effective to enhance SNR in the low spatial frequency region in OBF STEM across wide range of thickness.

## 4 Statistical Analysis

This study is based on theoretical calculations and multislice image simulations, and no statistical hypothesis testing was performed. The low‐dose images were generated by Poisson sampling from the expected electron counts calculated from the corresponding infinite‐dose datasets.

## 5 Conclusions

In this study, we explored how lens aberrations can improve the SNR in OBF STEM. We confirmed that defocus increases SNR, but its oscillating CTF makes the resultant images difficult to interpret. In contrast, introducing spherical aberration also increases SNR while keeping the CTF smooth, and thus, the image remains easy to interpret. For practical implementation, it is necessary to measure and control additional aberrations that are inevitably introduced when intentional aberrations are applied; we are developing such methods and will report them in a separate paper. Encouragingly, our recent progress suggests that 4D‐STEM–based aberration measurement can provide a practical route to such aberration control. The proposed method, combined with the aberration control technique, should enable efficient imaging of beam‐sensitive materials such as zeolites.

## Conflicts of Interest

Some of the authors are inventors of the OBF STEM technique and hold related patents.

## Supporting information




**Supporting File**: smtd70711‐sup‐0001‐SuppMat.pdf.

## Data Availability

Code used for the simulations in this study is publicly available at https://github.com/sigma‐users/Enhancing‐dose‐efficiency‐of‐OBF‐STEM‐by‐using‐phase‐shifted‐electron‐probe.

## References

[1] S. J. Pennycook and P. D. Nellist , Scanning Transmission Electron Microscopy: Imaging and Analysis (Springer Science & Business Media, 2011), 10.1007/978-1-4419-7200-2.

[2] R. F. Egerton , “Radiation Damage to Organic and Inorganic Specimens in the TEM,” Micron 119 (2019): 72–87, 10.1016/j.micron.2019.01.005. 30684768

[3] N. Dimova , R. Plackett , and D. Weatherill , “Measurement of the Resolution of the Timepix4 Detector for 100 and 200 keV Electrons for Transmission Electron Microscopy,” Nuclear Instruments and Methods in Physics Research Section A: Accelerators, Spectrometers, Detectors and Associated Equipment 1075 (2025): 170335, 10.1016/j.nima.2025.170335.

[4] P. Zambon , S. Bottinelli , and R. Schnyder , “KITE: High Frame Rate, High Count Rate Pixelated Electron Counting ASIC for 4D STEM Applications Featuring High‐Z Sensor,” Nuclear Instruments and Methods in Physics Research Section A: Accelerators, Spectrometers, Detectors and Associated Equipment 1048 (2022): 167888.

[5] N. Shibata , Y. Kohno , S. D. Findlay , H. Sawada , Y. Kondo , and Y. Ikuhara , “New Area Detector for Atomic‐resolution Scanning Transmission Electron Microscopy,” Journal of Electron Microscopy 59 (2010): 473–479, 10.1093/jmicro/dfq014. 20406732

[6] J. M. Rodenburg and R. H. T. Bates , “The Theory of Super‐Resolution Electron Microscopy via Wigner‐Distribution Deconvolution,” Philosophical Transactions of the Royal Society of London Series A: Physical and Engineering Sciences 339 (1992): 521–553.

[7] J. M. Rodenburg , B. C. McCallum , and P. D. Nellist , “Experimental Tests on Double‐Resolution Coherent Imaging via STEM,” Ultramicroscopy 48 (1993): 304–314, 10.1016/0304-3991(93)90105-7.

[8] A. M. Maiden and J. M. Rodenburg , “An Improved Ptychographical Phase Retrieval Algorithm for Diffractive Imaging,” Ultramicroscopy 109 (2009): 1256–1262, 10.1016/j.ultramic.2009.05.012. 19541420

[9] C. Ophus , “Four‐Dimensional Scanning Transmission Electron Microscopy (4D‐STEM): From Scanning Nanodiffraction to Ptychography and beyond,” Microscopy and Microanalysis 25 (2019): 563–582, 10.1017/S1431927619000497. 31084643

[10] T. J. Pennycook , A. R. Lupini , H. Yang , M. F. Murfitt , L. Jones , and P. D. Nellist , “Efficient Phase Contrast Imaging in STEM Using a Pixelated Detector. Part 1: Experimental Demonstration at Atomic Resolution,” Ultramicroscopy 151 (2015): 160–167, 10.1016/j.ultramic.2014.09.013. 25458189

[11] G. Li , H. Zhang , and Y. Han , “4D‐STEM Ptychography for Electron‐Beam‐Sensitive Materials,” ACS Central Science 8 (2022): 1579–1588, 10.1021/acscentsci.2c01137. 36589892 PMC9801507

[12] H. Sha , J. Cui , and J. Li , “Ptychographic Measurements of Varying Size and Shape Along Zeolite Channels,” Science Advances 9 (2023): adf1151, 10.1126/sciadv.adf1151. PMC10017048 36921047

[13] G. Li , M. Xu , and W.‐Q. Tang , “Atomically Resolved Imaging of Radiation‐Sensitive Metal‐Organic Frameworks via Electron Ptychography,” Nature Communications 16 (2025): 914, 10.1038/s41467-025-56215-z. PMC11750992 39837871

[14] K. Ooe , T. Seki , Y. Ikuhara , and N. Shibata , “Ultra‐High Contrast STEM Imaging for Segmented/Pixelated Detectors by Maximizing the Signal‐to‐Noise Ratio,” Ultramicroscopy 220 (2021): 113133, 10.1016/j.ultramic.2020.113133. 33181363

[15] K. Ooe , T. Seki , M. Nogami , Y. Ikuhara , and N. Shibata , “Dose‐Efficient Phase‐Contrast Imaging of Thick Weak Phase Objects via OBF STEM Using a Pixelated Detector,” Microscopy 74 (2025): 98–106, 10.1093/jmicro/dfae051. 39506558 PMC11957251

[16] T. Seki , N. Takanashi , and E. Abe , “Integrated Contrast‐Transfer‐Function for Aberration‐Corrected Phase‐Contrast STEM,” Ultramicroscopy 194 (2018): 193–198, 10.1016/j.ultramic.2018.08.008. 30170253

[17] T. Seki , K. Khare , and Y. O. Murakami , “Linear Imaging Theory for Differential Phase Contrast and Other Phase Imaging Modes in Scanning Transmission Electron Microscopy,” Ultramicroscopy 240 (2022): 113580, 10.1016/j.ultramic.2022.113580. 35908324

[18] K. Ooe , T. Seki , K. Yoshida , Y. Kohno , Y. Ikuhara , and N. Shibata , “Direct Imaging of Local Atomic Structures in Zeolite Using Optimum Bright‐Field Scanning Transmission Electron Microscopy,” Science Advances 9 (2023): adf6865, 10.1126/sciadv.adf6865. PMC10396294 37531431

[19] H. Yang , P. Ercius , P. D. Nellist , and C. Ophus , “Enhanced Phase Contrast Transfer Using Ptychography Combined With a Pre‐Specimen Phase Plate in a Scanning Transmission Electron Microscope,” Ultramicroscopy 171 (2016): 117–125, 10.1016/j.ultramic.2016.09.002. 27664566

[20] T. Seki , Y. Ikuhara , and N. Shibata , “Theoretical Framework of Statistical Noise in Scanning Transmission Electron Microscopy,” Ultramicroscopy 193 (2018): 118–125, 10.1016/j.ultramic.2018.06.014. 30005321

[21] M. J. Waters and J. Rondinelli , “MTD‐group/papuc: First Release of PAPUC,” Zenodo (2020), 10.5281/ZENODO.3688502.

[22] J. Madsen and T. Susi , “The abTEM Code: Transmission Electron Microscopy from First Principles,” Open Research Europe 1 (2021): 24, 10.12688/openreseurope.13015.1. 37645137 PMC10446032

[23] C. M. O'Leary , G. T. Martinez , E. Liberti , M. J. Humphry , A. I. Kirkland , and P. D. Nellist , “Contrast Transfer and Noise Considerations in Focused‐probe Electron Ptychography,” Ultramicroscopy 221 (2021): 113189. 33360480 10.1016/j.ultramic.2020.113189

