# Supplementary material for: Enhancing Dose Efficiency of Optimum Bright‐Field Scanning Transmission Electron Microscopy Using a Phase‐Shifted Electron Probe
Source: Small Methods. 2026 May 17;10(11):e01661. doi: 10.1002/smtd.202501661 (PMC13244290; doi:10.1002/smtd.202501661)
Supplement: Supplementary file 1 — Supporting File: smtd70711‐sup‐0001‐SuppMat.pdf. [file SMTD-10-e01661-s001.pdf]

Enhancing dose efficiency of OBF STEM by using phase shifted electron probe

Mitsutu Nogami[1], Takehito Seki[1,2]*, Kousuke Ooe[3,4], Naoya Shibata[1,4]*

1. Institute of Engineering Innovation, School of Engineering, The University of Tokyo, Tokyo, 113-8656, Japan
2. PRESTO, Japan Science and Technology Agency, Kawaguchi, 332-0012, Japan
3. School of Physics and Astronomy, Monash University, Clayton, Victoria, 3800, Australia
4. Nanostructures Research Laboratory, Japan Fine Ceramics Center, 2-4-1, Mutsuno, Atsuta, Nagoya, Aichi 456-8587, Japan

S1. Combined effect of spherical aberration and defocus

In the main text, we mainly discuss the effect of spherical aberration (Cs) on the SNR transfer function and OBF imaging while keeping defocus (Δf) close to zero. In practical operation, however, Cs and Δf usually coexist. Here we briefly examine their combined effect.

First, we calculated the SNR transfer function for a fixed spherical aberration of Cs = 180 μm at several defocus values, Δf = 0, 10, 30, and 60 nm (Fig. S1). The results show that a finite defocus slightly enhances the SNR transfer at the very lowest spatial frequencies. At the same time, the oscillations at intermediate spatial frequencies become stronger as Δf increases. The high-frequency region is unchanged.

Next, we performed multislice 4D-STEM simulations of FAU-type zeolite at a thickness of 10 nm. OBF images were reconstructed for two conditions: (i) Cs = 180 μm and Δf = 0, and (ii) Cs = 180 μm and Δf = 30 nm. For each condition, we generated images at infinite dose and at a low dose (Fig. S2). The two sets of images are very similar: the visibility of the framework and pore structure is essentially the same, and the additional defocus does not provide a clear improvement in the low-dose images.

These results indicate that, once a suitable Cs is introduced, adding defocus provides only a small gain at very low spatial frequencies while reintroducing oscillatory transfer. In practice, the main optimization can therefore focus on tuning Cs for low-frequency SNR enhancement, while keeping Δf close to zero to avoid unnecessary artifacts.

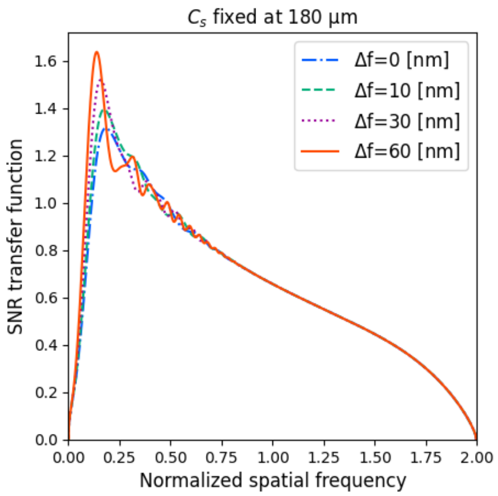

Fig. S1: SNR Transfer functions for thickness of 10 nm under various defocus values with spherical aberration fixed at Cs=180 um

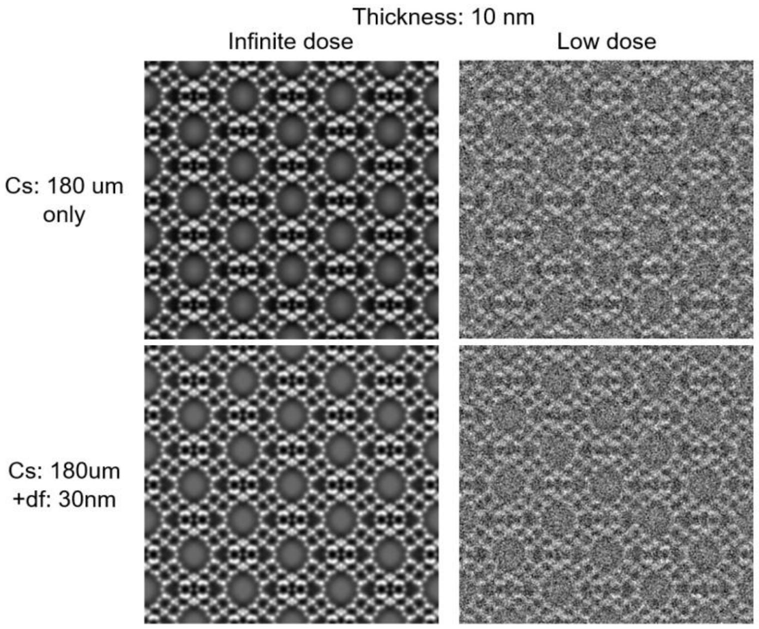

Fig. S2: Simulated OBF-STEM images of FAU zeolite (thickness 10 nm) under spherical aberration Cs = 180 μm. Images are shown for infinite-dose (left) and low-dose (right) conditions. The top row uses Cs only, while the bottom row uses Cs with additional defocus (Δf = 30 nm).

S2. Defocus dependence of pore-center contrast in OBF-STEM

To examine the effect of defocus on the pore-center contrast, we performed additional OBF image simulations for the same pore region at Δf = ±10, ±20, and ±30 nm, with the specimen thickness fixed at 10 nm (Fig. S3). As the absolute defocus increases, the apparent contrast at the pore center becomes systematically stronger. In parallel, oscillations in the phase contrast transfer function (PCTF) become more pronounced (Fig. S4). These results indicate that the pore-center signals originate from defocus-induced artifacts associated with the oscillatory PCTF.

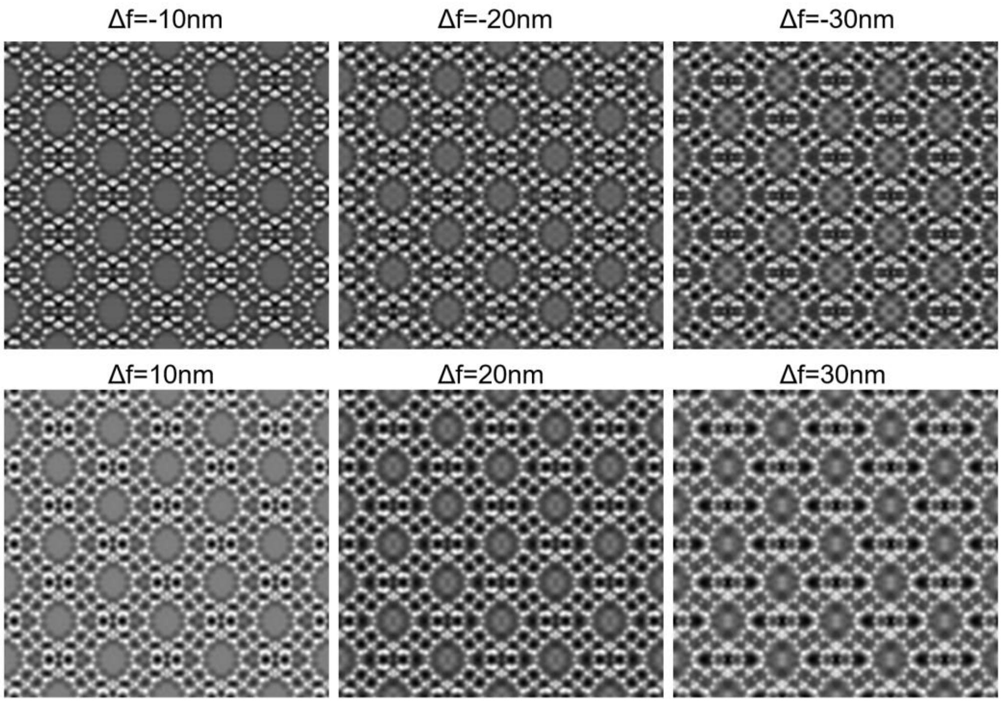

Fig. S3: Simulated OBF images with defocus values of Δf = ±10, ±20, and ±30 nm for with the thickness of 10 nm (infinite dose condition).

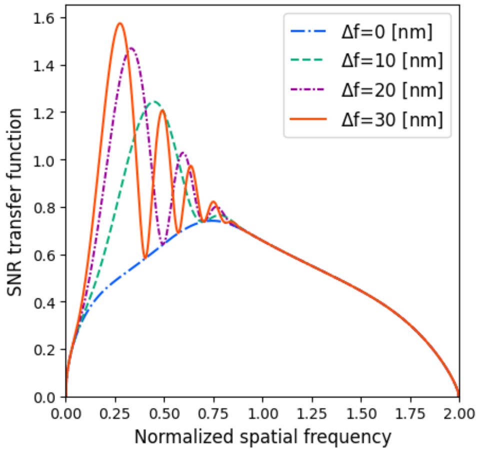

Fig. S4: SNR transfer functions with Δf = 10, 20, and 30 nm. The SNR transfer function is invariant under sign inversion.

S3. Comparison of tWPOA and multislice simulations for the G map

We computed the disk overlap patterns for FAU zeolite by multislice 4D-STEM simulations and by the tWPOA (Fig. S5). While some fine details differ, the main features of the G distribution are reproduced. This indicates that tWPOA provides a reasonable qualitative description within the thickness range considered in this work. Consistently, the multislice simulation results in Fig. 3 agree with the trends expected from the SNR transfer functions as discussed in main text.

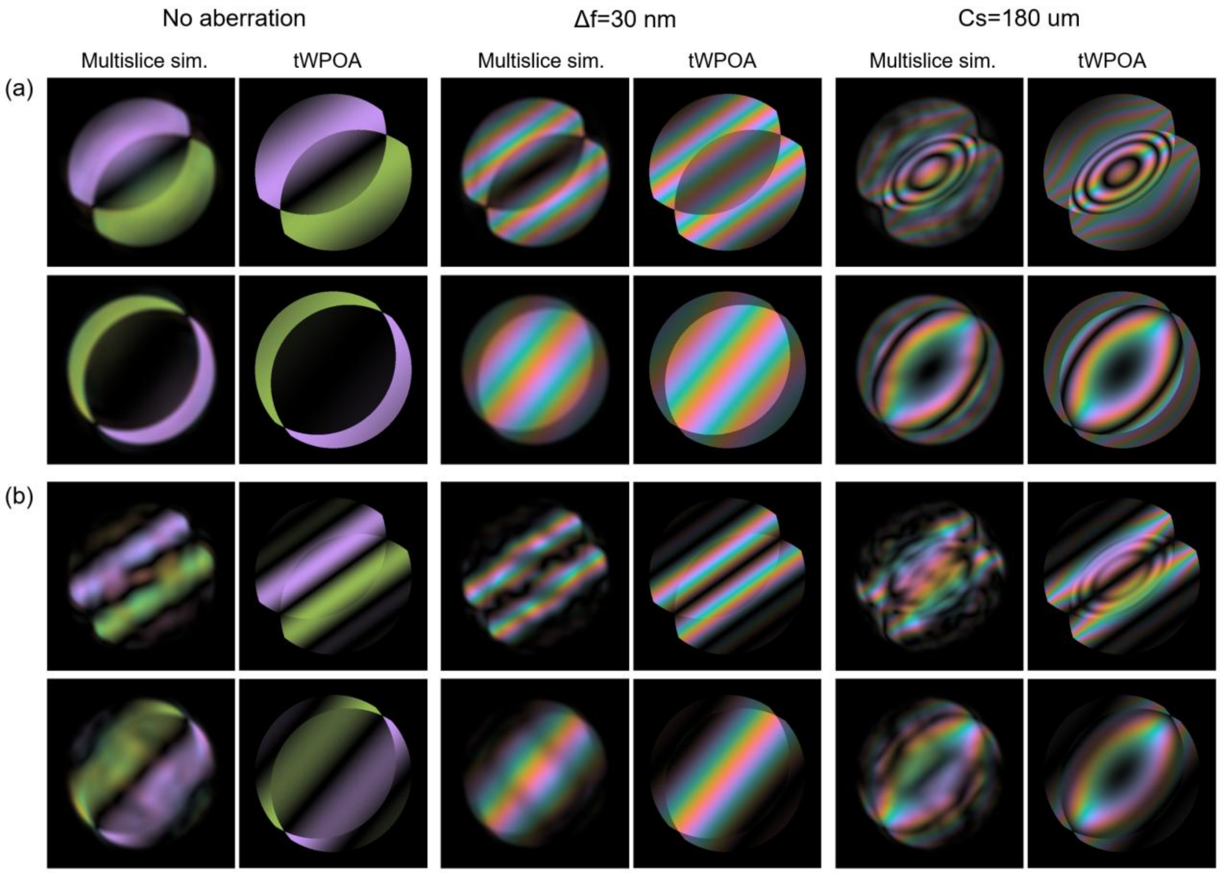

Fig. S5: Disk-overlap patterns for FAU zeolite calculated by multislice 4D-STEM simulations and by the tWPOA. Patterns are shown for specimen thicknesses of (a) 10 nm and (b) 30 nm. For each thickness, two representative spatial frequencies are shown (top and bottom rows) under three aberration conditions (no aberration, defocus Δf = 30 nm, and spherical aberration Cs = 180 μm). For each aberration condition, the left panel shows the multislice result and the right panel shows the corresponding tWPOA result.
